# Spatially selective p-type doping for constructing lateral WS_2_ p-n homojunction via low-energy nitrogen ion implantation

**DOI:** 10.1038/s41377-024-01477-3

**Published:** 2024-05-30

**Authors:** Yufan Kang, Yongfeng Pei, Dong He, Hang Xu, Mingjun Ma, Jialu Yan, Changzhong Jiang, Wenqing Li, Xiangheng Xiao

**Affiliations:** School of Physics and Technology, Key Lab of Artificial Micro- and Nano-Structures of Ministry of Education, Wuhan University, Wuhan, China

**Keywords:** Electronics, photonics and device physics, Optical materials and structures

## Abstract

The construction of lateral p-n junctions is very important and challenging in two-dimensional (2D) semiconductor manufacturing process. Previous researches have demonstrated that vertical p-n junction can be prepared simply by vertical stacking of 2D materials. However, interface pollution and large area scalability are challenges that are difficult to overcome with vertical stacking technology. Constructing 2D lateral p-n homojunction is an effective strategy to address these issues. Spatially selective p-type doping of 2D semiconductors is expected to construct lateral p-n homojunction. In this work, we have developed a low-energy ion implantation system that reduces the implanted energy to 300 eV. Low-energy implantation can form a shallow implantation depth, which is more suitable for modulating the electrical and optical properties of 2D materials. Hence, we utilize low-energy ion implantation to directly dope nitrogen ions into few-layer WS_2_ and successfully realize a precise regulation for WS_2_ with its conductivity type transforming from n-type to bipolar or even p-type conduction. Furthermore, the universality of this method is demonstrated by extending it to other 2D semiconductors, including WSe_2_, SnS_2_ and MoS_2_. Based on this method, a lateral WS_2_ p-n homojunction is fabricated, which exhibits significant rectification characteristics. A photodetector based on p-n junction with photovoltaic effect is also prepared, and the open circuit voltage can reach to 0.39 V. This work provides an effective way for controllable doping of 2D semiconductors.

## Abstract (web summary)

Low-energy ions implantation is used for spatially selective p-type doping to construct lateral p-n homojunction. The as-fabricated lateral WS_2_ p-n homojunction is applied in self-powered photodetector.

## Graphical abstract

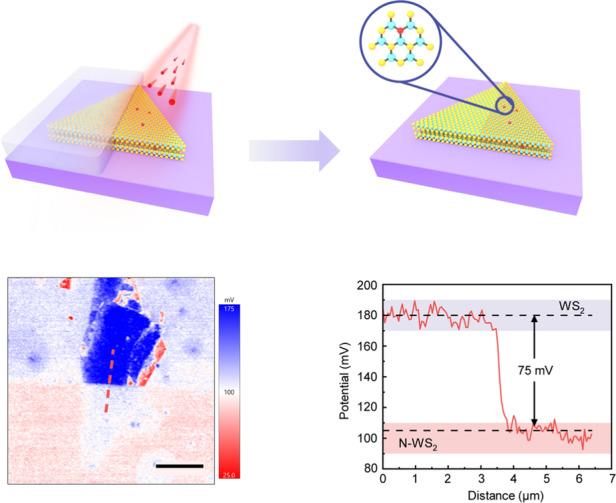

## Introduction

Two-dimensional (2D) metal chalcogenides (MDs) have significant potential for application in the next-generation high-performance photoelectric devices owing to their wide tunable bandgap, atomically sharp interface and strong light-matter interaction^1–4^. However, researchers are not content with the limited physical properties of single material and are dedicated to constructing various kinds of p-n junctions to design more functional devices. Previous researches have demonstrated that vertical p-n junction can be prepared simply by vertical stacking two materials with different conductivity types regardless of lattice mismatch^5,6^. However, the van der Waals (vdWs) gap existing between the interface will decrease the carrier mobility. Furthermore, the stacking process inevitably introduces impurities which can also reduce the device performance. In contrast, a lateral p-n junction based on two materials is seamlessly connected by covalent bond, thereby ensuring a clean junction interface.

Till now, great efforts have been made in the matters of chemistry and physics to fabricate 2D lateral p-n junction. For chemical strategies, chemical vapor deposition (CVD) epitaxial growth is pollution-free but the fabrication progress is complicated^7,8^. Chemical solvent doping is a useful method, but its lack of stability is a concern^9,10^. For pure physical methods, O_2_ or N_2_ plasma treatment has been frequently employed for spatially selective p-type doping of 2D MDs^11–13^. Despite plasma doping avoids impurity pollution, the doping elements are usually limited to gas and the implantation depth and concentration are lack of control. Ion implantation technique, a doping method to construct p-n junction in the traditional semiconductor industry, has the merits of controllable doping concentration and depth, uniform doping area and non-polluting doping process. It is worth noting that almost all elements, whether gases or metals, can be implanted into the target materials^14^. Therefore, it has been widely applied in 2D materials^15–19^, e.g., defects modulation by ion beam^20,21^, material growth control by substrate implantation^22,23^, and more. However, it is difficult to directly modulate the electrical and optical properties of 2D materials by conventional ion implantation. This is because energetic ions (tens of keV) will cause damage or even penetrate the atomically thin 2D materials during the implantation process. Here, we have developed a low-energy ion implantation system that reduces the implanted energy to 300 eV. Low-energy ion implantation method inherits all the merits of the traditional ion implantation technique. It has a lower ion energy and shallower implantation depth^24–27^. It is worth noting that the implantation depth here refers to the probability density distribution of implanted ions in the target materials. Bangert et al. for the first time experimentally demonstrate that low-energy nitrogen (N) and selenium (Se) ions can be effectively implanted into graphene and 2D MDs (MoS_2_), respectively, without causing high-density defects^28^. Although, a few groups are applying low-energy ion implantation technology to 2D materials, they mainly focus on the effects of low-energy ion implantation on 2D materials in PL^29^, metal-semiconductor contact^30^, TEM^28^, and defect modulation^31^. To date, there is a dearth of research investigating the utilization of low-energy ion implantation technology for achieving spatially selective p-type doping on 2D materials, thereby completely reversing their conductivity types and constructing lateral p-n homojunctions.

In this work, the doping of N-ions into few-layer WS_2_ flakes was achieved through low-energy ion implantation. Notably, nitrogen serves as an effective p-type dopant by facilitating hole injection into the intrinsic n-type WS_2_. By modulating the implantation dose, the conductivity type of the WS_2_ flake could be transformed from n-type to bipolar or even p-type conduction. After rapid thermal annealing, the current on/off ratio and hole mobility of N-implanted WS_2_ were greatly enhanced. This strategy has also been demonstrated applicable to other MDs such as WSe_2_, SnS_2_, and MoS_2_. In combination with electron beam lithography (EBL), the lateral WS_2_ p-n homojunction was fabricated. Based on this, a photodetector based on p-n junction with photovoltaic effect was also obtained. It possessed an open circuit voltage of 0.39 V, a responsivity of 35 mA W^−1^, a detectivity of 9.8 × 10^10^ Jones, and a rise/fall time of 5.6 ms/4 ms. This work proposes a spatially selective doping method compatible with integrated circuits, which provides an effective way to directly modulate the performance of 2D MDs by low-energy ion implantation.

## Results

**Fig. 1:**
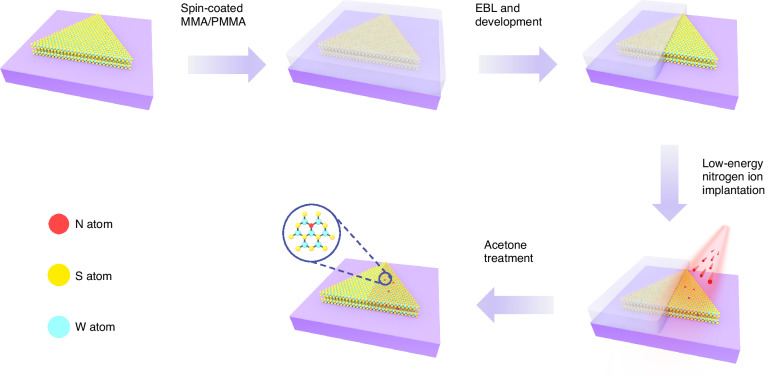
Experimental process diagrams for the fabrication of a lateral WS_2_ p-n homojunction realized by low-energy nitrogen ion implantation

Figure 1 illustrates the fabrication process of the lateral WS_2_ p-n homojunction realized by low-energy N-ion implantation. Initially, the WS_2_ flakes were mechanically exfoliated onto the Si/SiO_2_ substrates. Subsequently, a mask was created by spin-coating Methyl methacrylate (MMA) and poly (methyl methacrylate) (PMMA) on top of the flakes. Then, electron beam lithography (EBL) and development were carried out to reveal the selected region of the flake, while the remaining region was covered by MMA/PMMA. Whereafter, the low-energy nitrogen ions were successfully implanted into the uncovered WS_2_ flake. To calculate the stopping range of nitrogen ions in the WS_2_ flake, SRIM Monte Carlo simulations were performed. It was worth noting that the implantation energy was 300 eV (with a projected range of 1.8 nm), which ensured that the nitrogen dopants could remain in the lattice of WS_2_ flake (Fig. S1). After acetone treatment, the seamless lateral WS_2_ p-n homojunction was obtained.

**Fig. 2:**
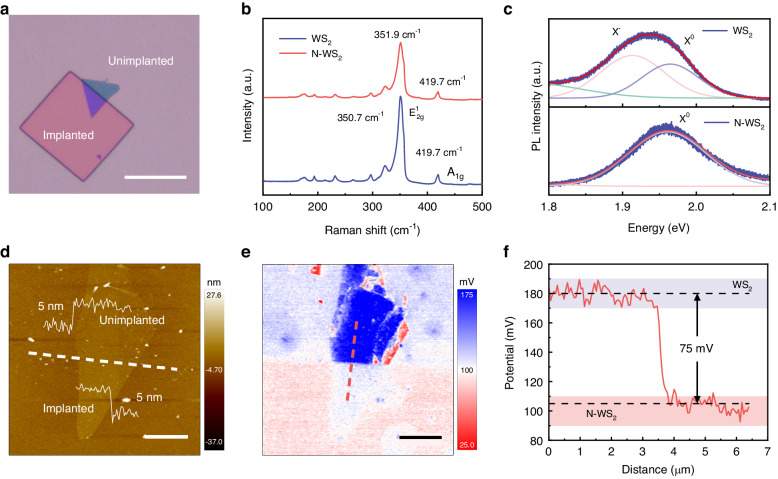
Material characterizaiton of pristine WS_2_ and N-WS_2_. **a** OM image of lateral WS_2_ p-n homojunction. The region within the square frame is the N-ions implanted region. The remaining region are unimplanted region. The scale bar is 20 μm. **b** Raman spectra and (**c**) PL spectra of pristine WS_2_ (blue curve) and N-WS_2_ (red curve) on the same sample. **d** AFM image of the implanted region and unimplanted region on the same sample, among the white dotted line is the dividing line. The height profiles insert show that the WS_2_ flake thickness in both regions are ~5 nm. **e** The contrastive KPFM mapping of the WS_2_ lateral p-n homojunction. The scale bar is 3 μm. **f** The surface potential difference of WS_2_ lateral p-n homojunction marked with the red line in **e**. The difference value is ~75 mV

The optical microscope (OM) image (Fig. 2a) indicates that the doped region maintains good morphology without visible damage. Raman spectra are carried out to further study the influence of the low-energy N-ion implantation on the lattice structure. As plotted in Fig. 2b, it is observed that the Raman characteristic peak positions of pristine WS_2_ locate at 350.7 cm^−1^ (the in-plane vibrational phonon mode of E^1^_2g_) and 419.7 cm^−1^ (the out-of-plane vibrational phonon mode of A_1g_), consistent with previous reports^32^. Whereas, the E^1^_2g_ peak of N-WS_2_ (WS_2_ flake treated with low-energy N-ion implantation) shows a slight blue shift about one wavenumber compared to the pristine sample. It is inferred that the doped nitrogen atom substitutes the position of sulfur atom and forms a shorter W-N bond (W-N bond is 0.2018 nm, W-S bond is 0.2416 nm)^11,13,33,34^. This may be considered to introduce the compressive strain in the crystal lattice^35^. The slightly decreased peak intensity indicates that the crystallization quality decreases slightly after the N-ion implantation process. Moreover, two small and sharp peaks have appeared at 148 cm^−1^ (Fig. S2a) and 384 cm^−1^ (Fig. S2b) after N-ion implantation treatment, which have been denoted as ZA(M) and A_2_’(M) phonon modes^36^. This result can be ascribed to the resonance Raman effect caused by external substitution doping^32,37,38^, which indicates a successful nitrogen doping. After the rapid thermal annealing process, both the E^1^_2g_ peaks of N-WS_2_ and the pristine display enhancement (Fig. S3). One of the possible reasons is that the rapid thermal annealing promotes the repairment of the lattice damage, and it improves the crystallization quality of the implanted sample. Subsequently, the photoluminescence (PL) spectra of the materials are also investigated before and after the N-ion implantation process.

As shown in Fig. 2c, the PL peak energy position of pristine WS_2_ is located at ~1.93 eV, while N-WS_2_ shows the blue shift to ~1.96 eV. We utilize Lorenz peak decomposition to analyze the reasons. The upper part of Fig. 2c shows that the PL peak of pristine WS_2_ is composed of two excitonic emission peak: the negative trions (X^−^) (lower energy) results from unintentional electrons doping provided by sulfur vacancy and the neutral excitons (X^0^) (higher energy) originates from the intrinsic carrier recombination^39,40^. The PL peak of pristine WS_2_ is dominated by X^−^ excitonic emission due to unintentional electron doping provided by the sulfur vacancy defects^39^. In N-WS_2_, the doped nitrogen act as acceptors to introduce a large number of holes and deplete the residual electrons to prevent the formation of X^−^. Thus, the PL peak of N-WS_2_ is dominated by switching to X^0^ recombination^7^, resulting in the peak blue shift as shown in the bottom part of Fig. 2c. Ion implantation is a lossy doping approach, which can degrade the crystallization quality and induce some defects to trap carriers to form nonradiative recombination centers resulting in the sharp decrease of PL intensity^36^. With the increase of N-ions implantation dose, the PL intensity continues to decrease, and eventually quenches (Fig. S4). After annealing treatment, the PL intensities (Fig. S5) of both two samples are slightly enhanced, which can be explained by the following reasons: (1) removal of the residual organic matter and absorbents on the surface; (2) repair of the damage lattice; (3) partial repair of the non-radiative recombination centers induced by ion bombardment. Atomic force microscopy (AFM) is utilized to measure the surface morphology and the thickness of the as-fabricated lateral WS_2_ p-n homojunction. In Fig. 2d, the AFM image reveals the flat surface throughout the domain. Meanwhile, the sample thickness of the N-implanted region is measured to be ~5 nm (~7 layers), which is about the same as the unimplanted region, indicating that the low-energy N-ions implantation does not reduce the thickness of the WS_2_ flakes. This result excludes that the obtained p-type WS_2_ is caused by material thickness change and verifies that the N-ions implantation is the cause of the obtained p-type WS_2_^41^. Kelvin probe force microscopy (KPFM) is also implemented to investigate the potential change of WS_2_ flake before and after the N-ions implantation (Note S1). The contrastive KPFM mapping is shown in Fig. 2e, the clear surface potential difference can be observed between the pristine WS_2_ and the N-WS_2_. Compare to pristine WS_2_, there is a significant decrease by 75 mV in surface potential for N-WS_2_ (Fig. 2f), corresponding to the increase in its work function. This further substantiates the p-type doping effect induced by N-ions implantation.

**Fig. 3:**
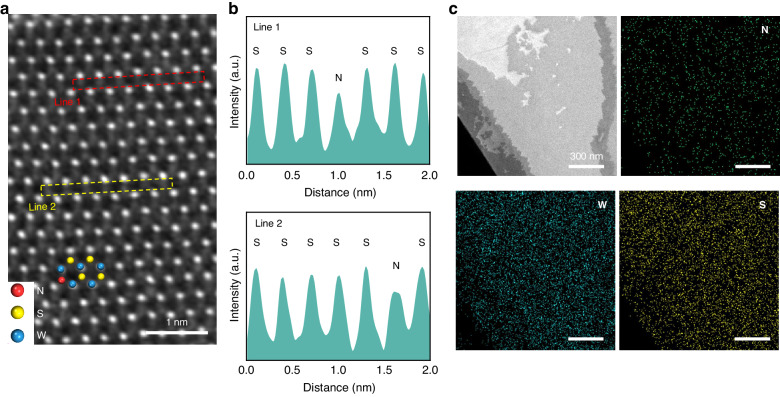
STEM characterization of N-WS_2_. **a** HAADF-STEM image of N-WS_2_. **b** The line profile intensity taken along the two lines of Line 1 and Line 2. **c** EDS element mapping of N-WS_2_ with even-distributed W, S, and N

In order to intuitively characterize the presence and distribution of doped nitrogen element, the N-WS_2_ is characterized by high-angle annular dark-field (HAADF) scanning transmission electron microscopy (STEM). In HAADF-STEM image, the brightness of atoms can be reflected by atomic number due to the Z-contrast, and atoms with higher atomic number produce brighter signal intensity^42^. Substitution of S by N is predicted to produce darker contrasts. As depicted in Fig. 3a, the hexagonal honeycomb structure of N-WS_2_ is clearly visible, and the darker point at the S site (the fourth atom in Line 1 and the sixth atom in Line 2) identified a N atom substituted to S site. The results are consistent with the theoretical calculation. Also, the line profile intensity along the direction indicated by Line 1 and Line 2 agrees well with the HAADF-STEM image (Fig. 3b: top, Line 1; bottom, Line 2), further confirming the substitution of S by N. As displayed in the energy dispersive spectroscope (EDS) elements mapping (Fig. 3c), W, S, and N elements are evenly distributed throughout the sample. Direct counting the number of N atoms substituted gives doping concentration of 2.26% at the dose of 1 × 10^15 ^ions cm^−2^ (Fig. S6).

**Fig. 4:**
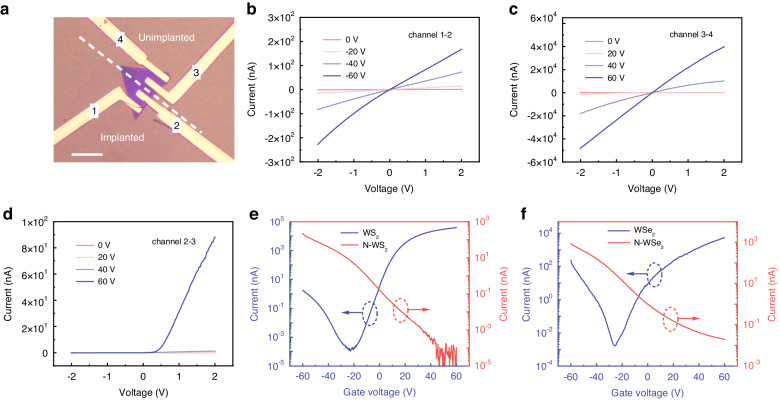
Electrical performance of pristine WS_2_ and N-WS_2_. **a** OM image of the as-fabricated WS_2_ devices. Below the white dotted line is the implanted region, while above the white dotted line is the unimplanted region. The sample is divided into three parts denoted as channel 1-2 (N-WS_2_), channel 2-3 (lateral WS_2_ p-n homojunction), and channel 3-4 (pristine WS_2_). The scale bar is 10 μm. **b** Output characteristic curves of N-WS_2_ FET at gate voltage sweep from 0 V to −60 V with −20 V step. Output characteristic curves of (**c**) WS_2_ FET and (**d**) lateral WS_2_ p-n homojunction FET at gate voltage sweep of 0 V to 60 V with 20 V step. **e** Transfer characteristic curves of WS_2_ and N-WS_2_ (with implantation dose of 1 × 10^14 ^ions cm^−2^) FETs at bias of 1 V in logarithmic scale. **f** Transfer characteristic curves comparison of WSe_2_ (blue curve) and N-WSe_2_ (red curve) FETs at bias of 1 V in logarithmic scale

To further explore the influence of low-energy N-ion implantation on the electrical properties of WS_2_, field-effect transistors (FETs) devices are fabricated. As illustrated in Fig. 4a, the sample is divided into three parts denoted as channel 1-2 (N-WS_2_), channel 2-3 (lateral WS_2_ p-n homojunction), and channel 3-4 (pristine WS_2_). The p-n junction is formed by a part of the implanted region and a part of the unimplanted region. Details of the device fabrication process are given in the “Materials and methods” section. Noteworthy, to lower the Schottky barrier height, Au electrodes with a higher work function (~5.1 eV) contact are used for N-WS_2_ contact, and Cr/Au electrodes with a lower work function (~4.6 eV) contact are used for WS_2_ contact. As shown in Fig. 4b, c, the output characteristic curves of both N-WS_2_ and pristine WS_2_ at different gate voltages exhibit linear curves. It can be ascribed to ohmic contact between the appropriately selected metal electrodes and material. The output characteristic curve of lateral WS_2_ p-n homojunction displayed in Fig. 4d presents that the junction has a significant rectification characteristic. The rectification ratio is approximately 550 under the bias of ±2 V and the gate voltage of 60 V. To evaluate the property of the p-n homojunction, the ideality factor (*n*) is calculated by Shockley diode equation (Note S2). The ideality factor of the p-n homojunction is about 1.46 at gate voltage of 60 V. This manifest that the current of the device is mainly dominated by the diffusion process rather than the recombination process under the forward bias^10^. It is an indication of the relatively high quality of the junction. Figure 4e plots the transfer characteristic curves of pristine WS_2_ (blue curve) and N-WS_2_ (red curve) with the implantation dose of 1 × 10^14 ^ions cm^−2^. The pristine WS_2_ exhibits typical n-type conduction due to the sulfur vacancies contributing more electrons^39^. As well as the current on/off ratio reaches 10^8^. Conversely, N-WS_2_ FET shows a remarkable p-type conduction, which is undoubtedly caused by the N-ion implantation. This is because the N-ion implantation injects a large number of holes, which causes the hole concentration to be much higher than the electron concentration, making the holes become the majority carriers in N-WS_2_. We calculated the carrier concentration to be 1.49 × 10^12 ^cm^−2^ for electron concentration and 4.09 × 10^12 ^cm^−2^ for hole concentration at zero gate voltage (Note S3). However, the carrier mobility and the current on/off ratio of N-WS_2_ are rather low just after implantation (Fig. S7). Actually, rapid thermal annealing is an effective way to activate the implanted ions, which leads to the obvious improvement of the carrier mobility. We obtained that the hole mobility is increased to 12.16 cm^2^ V^−1^ s^−1^ (Note S4), and the current on/off ratio is also raised to 3.9 × 10^6^ compared to the unannealed FET. Table 1 summarizes the electrical performance parameters obtained from other 2D MDs and doping techniques. The N-WS_2_ FET in this work is equal or superior to the devices acquired by other doping means.

**Table 1:** Summary of the electrical performance parameters based on other 2D MDs and doping techniques

Doping method	Dopant	Material	Type	On/off ratio	Hole mobility (cm^2^ V^−1^ s^−1^)	Refs.
MOCVD	Nb	MoS_2_	P-type	10^3^	1.43	Ref. ^7^
Solution	Sn	PtSe_2_	P-type	10^4^	0.26	Ref. ^9^
Plasma	N	WS_2_	P-type	/	0.53–1.7	Ref. ^11^
ALD+CVD	N	WS_2_	P-type	10^5^	18.8	Ref. ^34^
Plasma	O	WSe_2_	P-type	1.8 × 10^5^	/	Ref. ^51^
CVD	Nb	MoS_2_	P-type	10^3^	0.02	Ref. ^52^
CVD	V	WSe_2_	P-type	1.65 × 10^6^	1.91	Ref. ^53^
CVD	Nb	WS_2_	P-type	/	0.044	Ref. ^54^
Ion implantation	N	WS_2_	P-type	3.9 × 10^6^	12.16	This work

The improved performance of N-WS_2_ FET after rapid thermal annealing can be understood by the following, during the implantation process, energetic N-ions will generate a series of elastic collision in WS_2_, and transfer energy to the atoms of WS_2_ indiscriminatingly. The colliding atoms will be released from the original lattice sites when they gain enough energy. It’s worth noting that the atomic mass of the sulfur atom is less than that of the tungsten atom, and thus it is easier for sulfur atoms to be unbound during the implantation process. Consequently, N-ions implantation will cause a series of damages in the form of substitutions, interstitials and vacancies in the implanted WS_2_^43,44^. The carrier mobility and conductivity will decrease significantly. However, the rapid thermal annealing process can activate the implanted nitrogen to migrate to the substitutional sites and repair the crystal lattice damage of the material caused by the ion implantation process. As a result, the carrier mobility and conductivity of the sample are significantly improved compared to the unannealed samples. In addition, annealing can remove the organic matter adsorbed on the surface of the channel materials to improve the interface quality and reducing the carrier scattering. Moreover, it may improve the contact between electrodes and semiconductors, leading to the lowering of contact resistance, which is also benefit to the improvement of device performance^30^.

In order to investigate the influence of N-ions implantation doses on WS_2_ p-type doping level, the FETs devices are prepared with different implantation doses. Figures 4e and S8 show the transfer characteristic curves of WS_2_ FET (blue line) and N-WS_2_ FET (red line) at gradient doses respectively. The electrical properties are recorded after annealing. All pristine WS_2_ FETs exhibit typical n-type conduction. As the implantation dose increases, the p-type doping level becomes more significant. At the rather low dose of 1 × 10^12 ^ions cm^−2^, the N-WS_2_ FET still shows electron-dominant n-type conduction. But the threshold voltage is shifted to the right compared to the pristine WS_2_ FET, indicating the weak p-type doping caused by the N-ions implantation doses. When the dose is increased to 1 × 10^13 ^ions cm^−2^, the current corresponding to the negative gate voltage starts to increase and the current corresponding to the positive gate voltage decreases. This means that N-ions implantation can effectively improve the hole concentration and change the conductivity type from electron-dominant n-type to bipolar-type. When the dose is further increased to 1 × 10^14 ^ions cm^−2^, the hole concentration is much higher than the electron concentration. Therefore, the conduction polarity of the N-WS_2_ FET completely changes to the hole-dominant p-type conduction. At the higher dose of 1 × 10^15 ^ions cm^−2^, the off-state characteristic of N-WS_2_ FETs cannot be well regulated by the gate voltage. To sum up, we successfully realize the regulation of the conductivity type of WS_2_ by precisely changing the N-ion implantation doses. The implanted WS_2_ can sequentially transform the conductivity type from n-type to bipolar- or even p-type conduction. In order to assess the device uniformity of p-type doped WS_2_, we calculate the hole concentration and mobility of N-WS_2_ on the same device for ten cycles, as depicted in Fig. S9. The cycling performance of N-WS_2_ exhibits remarkable stability and remains unaffected by an increase in cycle numbers. Additionally, we calculate the hole concentration and mobility of N-WS_2_ across different devices, as illustrated in Fig. S10, the performance of different devices shows little difference. Subsequently, we verify the device stability of N-ion doping. After 3 months of vacuum preservation, the N-WS_2_ FET performance is slightly degraded and still exhibits p-type characteristic (Fig. S11). This means that low-energy ion implantation is a stable doping method for 2D materials.

**Fig. 5:**
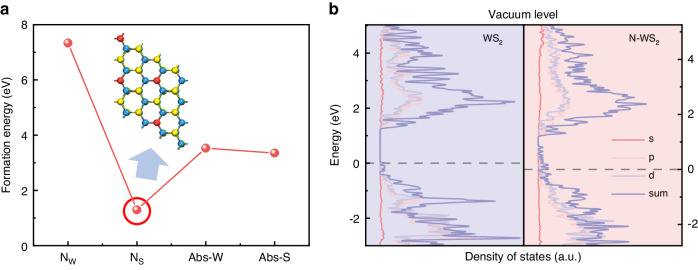
DFT calculation of formation energies and charge density of states of N-WS_2_. **a** Calculated formation energies of four possible models of N-doped sites in few-layer WS_2_. **b** TDOS and PDOS of pristine WS_2_ flake (left) and N-WS_2_ flake (right). The vacuum level is aligned and the Fermi level is set at 0 eV

To further understand our experimental result, density functional theory (DFT) is employed to explore the doping effect and electronic structure of N-WS_2_. We construct four hypothetically possible models of N-doped sites (Fig. S12), including substitutions (N_S_ and N_W_) and interstitials (Abs-W and Abs-S), and calculate their formation energy. More detailed modeling parameters are provided in “Materials and methods” section. As shown in the DFT calculations results, the substitution of N atom at S site (N_S_) has the lowest formation energy among the four possible doping models (Fig. 5a). The theoretical model of N_S_ matches the experimental observation results of HADDF-STEM (Fig. 3). The theoretical calculation results prove once again that the implanted N-ions primarily exist as substitution sites for S atoms in a few-layer WS_2_. Therefore, in the subsequent calculation of the electronic structure of N-WS_2_, we only consider the condition of N_S_. To reveal the reason for p-type doping, we have conducted calculations for the total density of states (TDOS) and partial density of the states (PDOS), as depicted in Figs. 5b and S13. According to the TDOS, after aligning the vacuum levels, it is clear that the Fermi level shifts toward the valence band maximum (VBM) after N-ions implantation. We also calculate the work functions of pristine WS_2_ and N-WS_2_. As shown in Fig. S14, the work function of WS_2_ exhibits an increase after N doping, which is consistent with the observed trend in KPFM measurements (Fig. 2e, f). The potential profiles obtained from KPFM demonstrate a significant decrease in surface potential for WS_2_ after N doping, corresponding to the observed increase in its work function. This further substantiates the p-type doping effect. As depicted in PDOS calculation (Fig. S13), the electronic states near the Fermi energy level of pristine WS_2_ are mainly caused by the hybridization between the W 5d orbitals and the S 3p orbitals. After N-ions implanted into the WS_2_ crystal, the impurity states emerge in close proximity to the Fermi level, resulting in shallow acceptor levels above the VBM in WS_2_. Notably, these impurity states predominantly originate from N 2p orbital contributions. All evidences prove that N doping can induce a p-type doping effect on WS_2_.

Finally, to verify the universality of the method to regulate the conductivity type of MDs via low-energy N-ion implantation, we extend this method to other 2D MD semiconductors. Figures 4f and S15 plot the transfer characteristic curves of pristine and N-doped FETs on WSe_2_, SnS_2_, and MoS_2_, respectively. Their conductivity types are significantly transformed from n-type to p-type conduction after low-energy N-ion implantation.

**Fig. 6:**
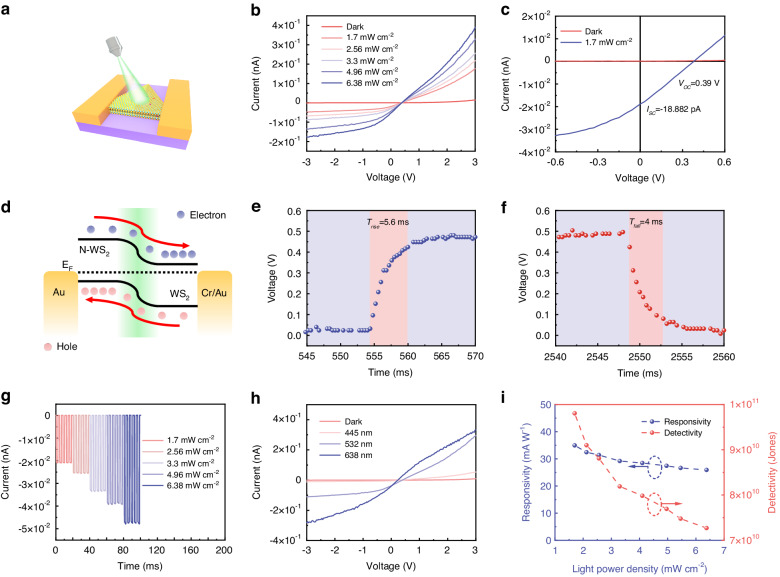
Photoelectric performance of the WS_2_ lateral p-n homojunction. **a** Schematic illustration of photodetector based on lateral WS_2_ p-n homojunction. **b** Output characteristic curves of lateral WS_2_ p-n homojunction under 532 nm illumination with different light power densities. **c** The magnified output characteristic curves of lateral WS_2_ p-n homojunction at gate voltage of 0 V under dark and 532 nm illumination (1.7 mW cm^−2^). **d** The energy band diagram of lateral WS_2_ p-n homojunction. **e** Rise and (**f**) fall time curve of transient photo-response under 532 nm illumination (4.96 mW cm^−2^) at bias of 1 V. **g** Transient photo-response of lateral WS_2_ p-n homojunction under 532 nm illumination with different light power densities at bias and gate voltage of 0 V. **h** Output characteristic curves of lateral WS_2_ p-n homojunction under different wavelength illumination of 445 nm, 532 nm, and 638 nm (4.1 mW cm^−2^). **i** Light power density dependence relation curves of responsivity and detectivity

In recent years, photodetectors without external power supply have attracted great attention. Because they can reduce energy consumption. It has great application prospects in optical communication, infrared detection and so on^45^. The p-n junction is the best candidate. Because it has a natural built-in electric field, which can promote the separation of electron-hole pairs and enhance the optical properties of 2D MDs materials. Based on this, we fabricate a photodetector based on the lateral WS_2_ p-n homojunction. Figure 6a shows the schematic illustration of the photodetector based on lateral WS_2_ p-n homojunction. The detailed device fabrication process is described in the “Materials and methods” section. Figure 6b shows the output characteristic curves before and after 532 nm laser illumination, the low dark current is 14.57 pA at 3 V bias without gate voltage. Under laser illumination, the current increases obviously and rises with the increase of light power density. Increasing the light power density can increase the number of photogenerated carriers, resulting in higher photocurrents^46^. The photodetector shows apparent photovoltaic effects under the 532 nm laser of 1.7 mW cm^−2^ with an open circuit voltage of 0.39 V and a short circuit current of −18.88 pA (Fig. 6c). Figure 6d illustrates the energy band diagram of lateral WS_2_ p-n homojunction under illumination which can explain the change of the photo-response. As can be seen from the surface potential measured by KPFM (Fig. 2e, f), there exists a built-in electric field pointed from WS_2_ to N-WS_2_ at the depletion region to prevent the infinite diffusion of the intrinsic carrier. When the device exposed to light, the photogenerated carriers will be separated by the built-in electric field. The photogenerated electrons drift to the WS_2_ region while the photogenerated holes drift to the N-WS_2_ region, generating a photocurrent. As shown in Fig. 6e, f, the response speed of the photodetector is expressed by the rise time and fall time, which calculated as the time difference value between 10% (90%) and 90% (10%) of the high level^47^. It can be deduced that the rise and fall times are 5.6 ms and 4 ms at bias of 1 V. The transient photo-response is studied under the periodic laser pulse of 2 s. Figure 6g depicts optical switching behavior of the photodetector measured at zero gate voltage and zero bias. When the laser is turned on, the current rises rapidly and remains stable, then falls rapidly when the laser is turned off. The on-off state can switch rapidly and the current can still remain stable after several cycles indicating that the high light current is contributed by the photogenerated carriers rather than the heating effect caused by laser^48^. Under varying light power densities, the photo-response current raises as the light power density increases. To investigate the capability of the photodetector to detect different wavelength lasers, three different wavelength lasers of 445 nm, 532 nm, and 638 nm were employed. Figure 6h plots the output characteristic curve of the three laser sources mentioned above at the light power density of 4.1 mW cm^−2^. The longer the incident wavelength, the higher the current generated. As the wavelength of laser light increases, the energy of photons decreases due to their inverse relationship. Consequently, when maintaining a constant light power density over a given time period, an increase in photon generation occurs as a result of lower photon energy, leading to an amplified photocurrent^49^. As depicted in Fig. S16, the transient photo-response diagram of different wavelength lasers (4.1 mW cm^−2^) shows that the photodetector exhibits stable switching behavior under different wavelength lasers illumination.

The parameters used to evaluate the performance of the photodetector are mainly responsivity (*R*), detectivity (D^*^) and external quantum efficiency (*EQE*). *R* is described as the ability of the photon to generate electron-hole pairs. It is calculated as $R(A{{W}}^{-1})=\frac{{I}_{{ph}}}{\rho S}$, where ${I}_{{ph}}$, $\rho$ and *S* are expressed as photogenerated current, light power density and effective illumination area, respectively^47^. Moreover, D^*^ is defined as the ability to detect weak light, i.e., ${D}^{* }\left({Jones}\right)=\,\frac{R\sqrt{S}}{\sqrt{2e{I}_{{dark}}}}$, where *e*, *R* and ${I}_{{dark}}$ are the electron charge, the responsivity and the dark current^47^. The *EQE* is expressed as the ratio of the number of electrons generated by the incident photon to the number of the incident photons per unit time. It is given by the equation, ${EQE}\left( \% \right)=\frac{{Rhc}}{\lambda e}\times 100 \%$, where *R*, *h*, *c*, $\lambda$ and *e* are the responsivity, Planck’s constant, light speed, incident wavelength and electron charge, respectively^47^. Figures 6i and S17 display the light power density dependence relation of *R*, D^*^, and *EQE*. With the increase of the light power density from 1.7 mW cm^−2^ to 6.38 mW cm^−2^, *R*, D^*^, and *EQE* show a decreasing trend. This result is related to the increasing recombination probability of photocarriers at increasing light power density^50^. Under the zero bias, the maximum *R*, D^*^, and *EQE* values reach to 35 mA W^−1^, 9.8 × 10^10^ Jones and 8.17%, respectively (532 nm laser of 1.7 mW cm^−2^). In addition, the *R*, D^*^, and *EQE* curves of the photodetector at different laser wavelengths are shown in Fig. S18. It can be discovered that the value of *R*, D^*^, and *EQE* reach the highest under the 532 nm laser. Thereby, the main performance of the photodetector is measured under the 532 nm laser.

## Discussion

In conclusion, we have demonstrated a strategy for selective p-type doping via low-energy N-ion implantation. Low-energy N-ions of 300 eV have been implanted into few-layer WS_2_ flakes, which ensure the nitrogen dopants remain in the lattice of WS_2_ flake. By accurately controlling the N-ions implantation doses, we successfully control the transformation of the n-type semiconductor WS_2_ into the bipolar- or even the p-type semiconductor. After rapid thermal annealing, the current on/off ratio and hole mobility of N-doped WS_2_ greatly enhanced. Moreover, this strategy is also applicable to several other MDs semiconductors including WSe_2_, SnS_2_, and MoS_2_. Based on the lateral WS_2_ p-n homojunction, the photodetector has been obtained. In this work, a purely physical method of low-energy ion implantation is proposed to realize selective p-type doping on MDs. By replacing the high-energy ion source with a lower-energy source, the performance of 2D materials can be directly and accurately modulated.

## Materials and methods

### Samples Preparation

High-quality few-layer WS_2_, WSe_2_, SnS_2_, and MoS_2_ flakes were mechanically exfoliated from their bulk crystal (Shanghai Onway Technology Co., Ltd.) and transferred onto 300 nm SiO_2_/Si substrates. Methyl methacrylate (MMA) and polymethyl methacrylate (PMMA) were spin-coated at 600 rpm for 6 s and 4000 rpm for 60 s to cover the flakes, and then baked at 120 °C for 1 min and 5 min, respectively. Electron beam lithography (EBL) and the following development were carried out to expose the selected region of the flakes, while the remaining region was covered by MMA/PMMA. Subsequently, the low-energy ions implantation technology was employed to implant N-ions into the selected region of WS_2_ flakes. After acetone treatment, a seamless lateral WS_2_ p-n homojunction was obtained.

### Low-energy ion implantation process

A low-energy ion implantation system with an adjustable ion energy of 300 eV–1.5 keV was used to implant nitrogen ions into 2D materials. When the vacuum was pumped below 6 × 10^−4 ^Pa, nitrogen gas was introduced into the vacuum chamber, and stabilized the pressure in the chamber at 1.2 × 10^−2 ^Pa. Subsequently, the nitrogen ions were extracted after ionization and screening processes, then the nitrogen ions were then implanted into the 2D material within a vacuum chamber using an accelerating voltage of 300 V.

### Device fabrication

The FET devices were fabricated by EBL and thermal evaporation. MMA and PMMA were spin-coated to cover the lateral WS_2_ p-n homojunction, using the same parameters as mentioned above. EBL was used to prepare the electrode patterns, including the regions of WS_2_, N-WS_2_, and lateral p-n homojunction on the same sample. The thermal evaporation was used for depositing the metal contact electrodes with 15 nm Cr and 50 nm Au for WS_2_ (electrode 3 and 4 in Fig. 4a) and 65 nm Au for N-WS_2_ (electrode 1 and 2 in Fig. 4a). After the device were prepared, rapid thermal annealing at 400 °C for 5 min was performed to improve the device performance.

### Density functional theory calculation

Density functional theory (DFT) calculations were performed by using CASTEP code package. The electronic exchange-correlation potential was carried out using the Perdew–Burke–Ernzerhof (PBE) functional of the generalized gradient approximation (GGA) and the ultrasoft pseudopotentials. The Brillouin zone integration was sampled and calculated by using a 1 × 2 × 1 Monkhorst-Pack k-point mesh. The kinetic energy cut-off was set as 500 eV for the plane-wave basis set. Van der Waals interactions were taken into account using the DFT dispersion (DFT-D) correction. To optimize the geometry, the convergence tolerances were set to be 5 × 10^−6^ eV energy per atom, 5 × 10^−4^ Å maximum displacement, and 0.01 eV Å^−1^ maximum force.

A four-layer WS_2_ (100) plane supercell (W_32_S_64_) was first constructed with a vacuum region of 15 Å along the z-axis for calculation. Additionally, considering that we mainly used ion implantation to modify the surface of WS_2_, the WS_2_ with two layers on the upper surface was considered to achieve N substitution doping and surface adsorption.

### Device characterization

#### Morphology and composition characterization

The optical microscopy images were obtained by an optical microscope (OLYMPUS BX51). Raman and PL spectra were characterized by the confocal Raman and Photoluminescence spectroscopy (LabRAM HR Evolution of HORIBA) with a 532 nm laser at room temperature. Atomic force microscopy (Jupiter XR) was used to measure the thickness of the materials. Kelvin probe force microscopy (Jupiter XR) was used to measure the surface potential of lateral p-n junction. High-angle annular dark-field (HAADF) scanning transmission electron microscopy (STEM) image and energy-dispersive spectrometer (EDS) mapping were carried out by JEM-ARM200CF. The measurements of electrical and optical properties were measured by the semiconductor device parameter analyzer instrument (TOSTAR 5514B) and vacuum probe station (Lake Shore, TTPX). The properties of the photodetector were measured under 445 nm, 532 nm, and 638 nm laser.

### Supplementary information

Supplementary information
