# Supplementary material for: Spatially selective p-type doping for constructing lateral WS2 p-n homojunction via low-energy nitrogen ion implantation
Source: Light Sci Appl. 2024 May 30;13:127. doi: 10.1038/s41377-024-01477-3 (PMC11143290; doi:10.1038/s41377-024-01477-3)
Supplement: Supplementary file 1 — Supplementary information [file 41377_2024_1477_MOESM1_ESM.docx]

**Supplementary Information for**

**Spatially selective p-type doping for constructing lateral WS_2_ p-n homojunction via low-energy nitrogen ion implantation**

Yufan Kang^1^, Yongfeng Pei^1^, Dong He^1^, Hang Xu^1^, Mingjun Ma^1^, Jialu Yan^1^, Changzhong Jiang^1^, Wenqing Li^1^*, and Xiangheng Xiao^1^*

^1^ School of Physics and Technology, Key Lab of Artificial Micro- and Nano-Structures of Ministry of Education, Wuhan University, Wuhan 430072, China

E-mail: wenqing_li@whu.edu.cn, xxh@whu.edu.cn


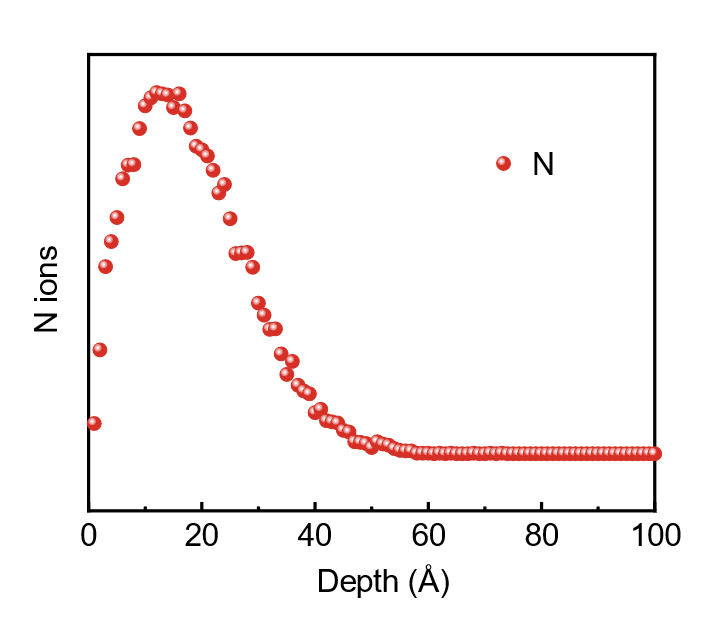


**Fig. S1** Depth ion distribution of nitrogen ions implanted in WS_2_ flake with 300 eV simulated by SRIM


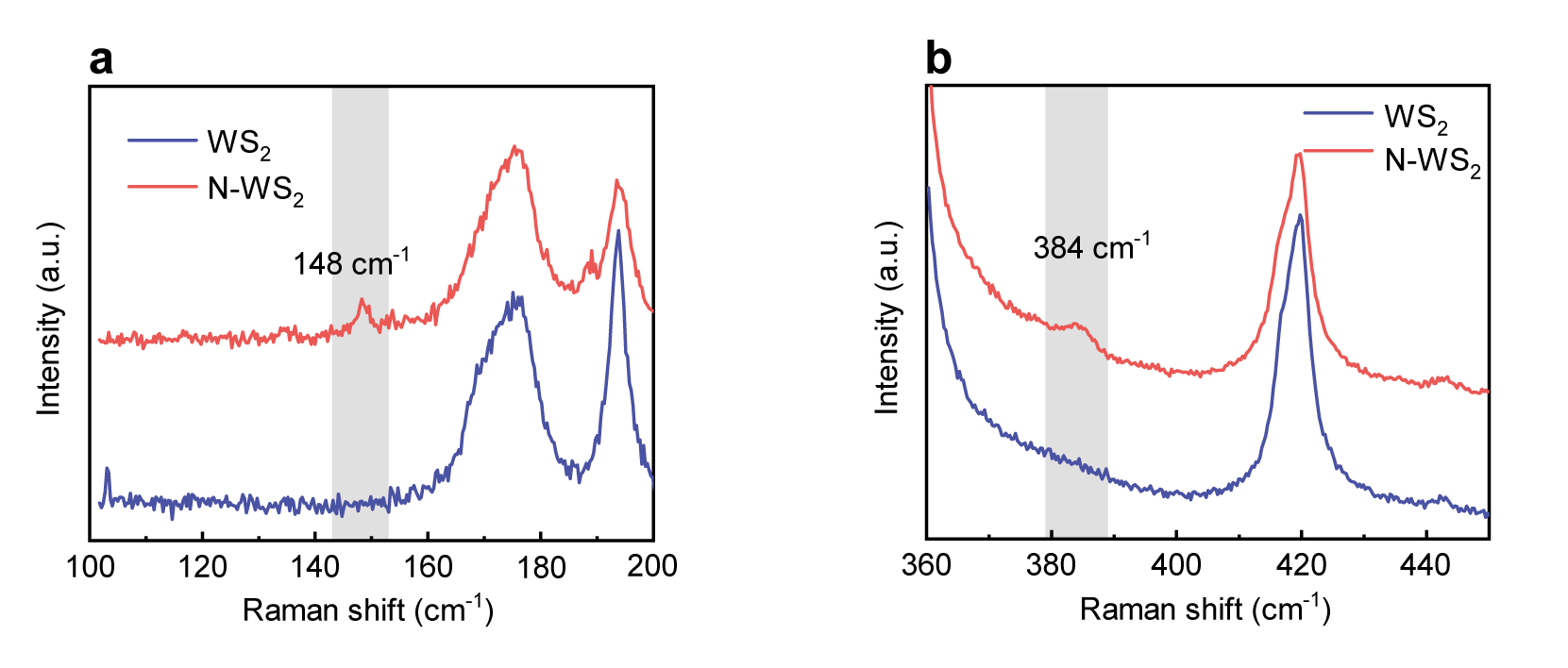


**Fig. S2** The magnified Raman spectra of the position locating at **(a)** 148 cm^-1^ and **(b)** 384 cm^-1^


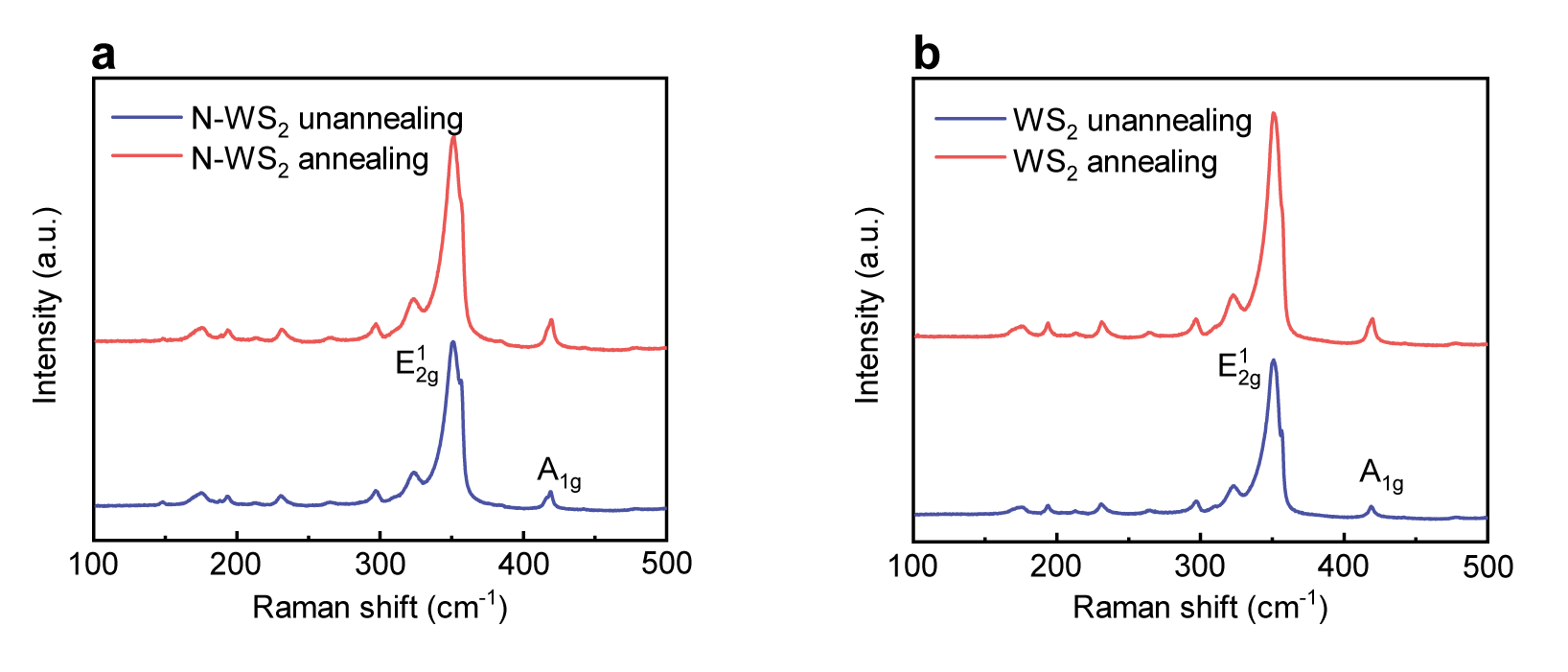


**Fig. S3** Raman spectra of **(a)** N-WS_2_ and **(b)** Pristine WS_2_ before and after rapid thermal annealing


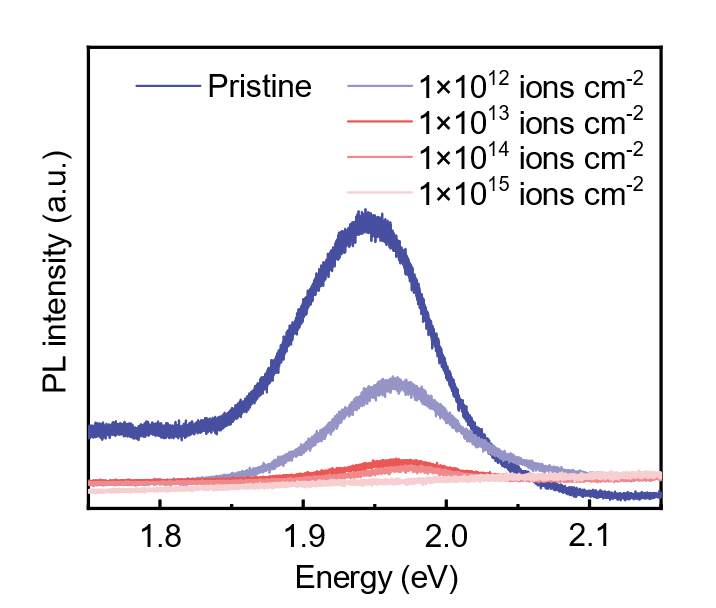


**Fig. S4** PL spectra of the same WS_2_ sample with different implantation doses


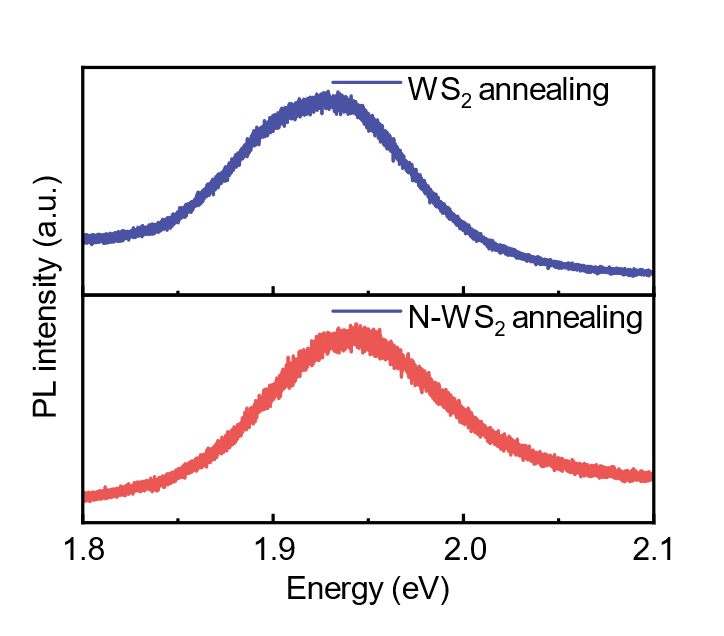


**Fig. S5** PL spectra of pristine WS_2_ and N-WS_2_ after annealing


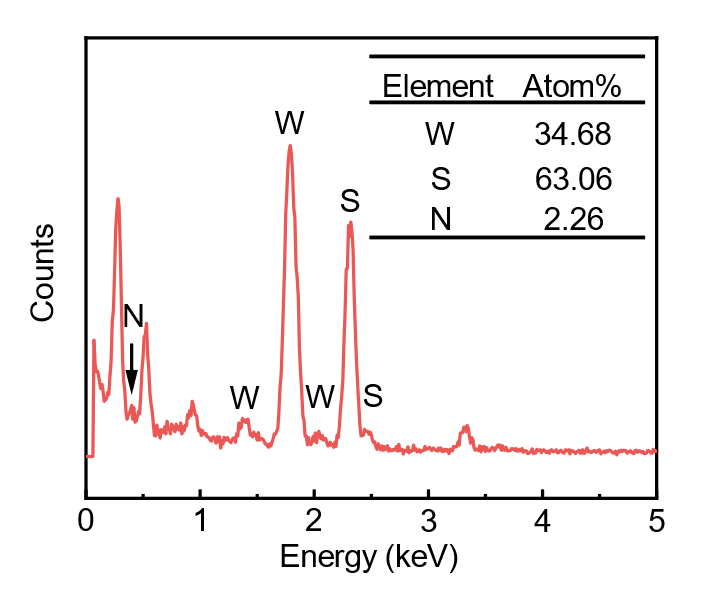


**Fig. S6** EDS data of few-layer N-WS_2_ flake at N-ions implantation dose of 1×10^15^ ions cm^-2^


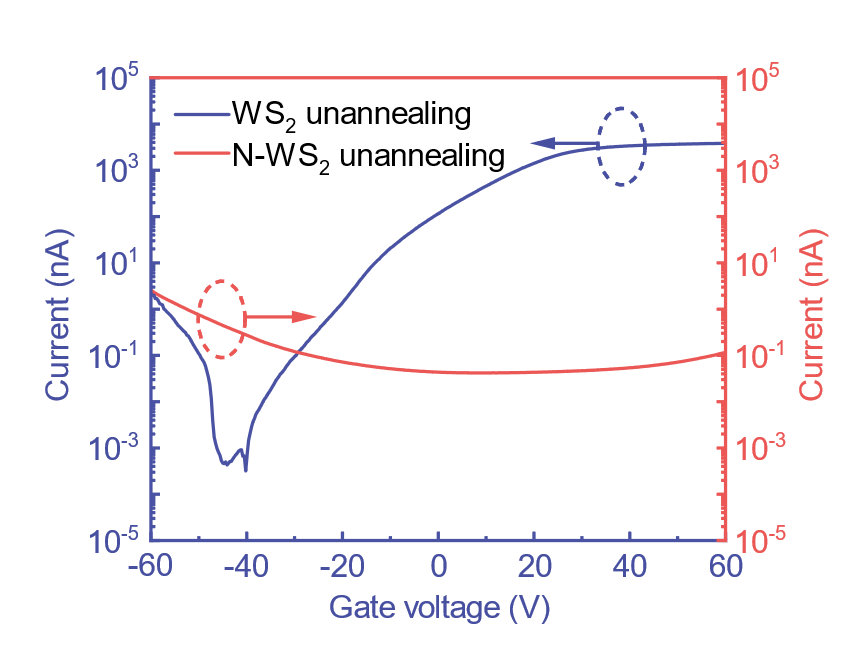


**Fig. S7** Transfer characteristic curves of WS_2_ and N-WS_2_ before annealing at the bias of 1 V in logarithmic scale


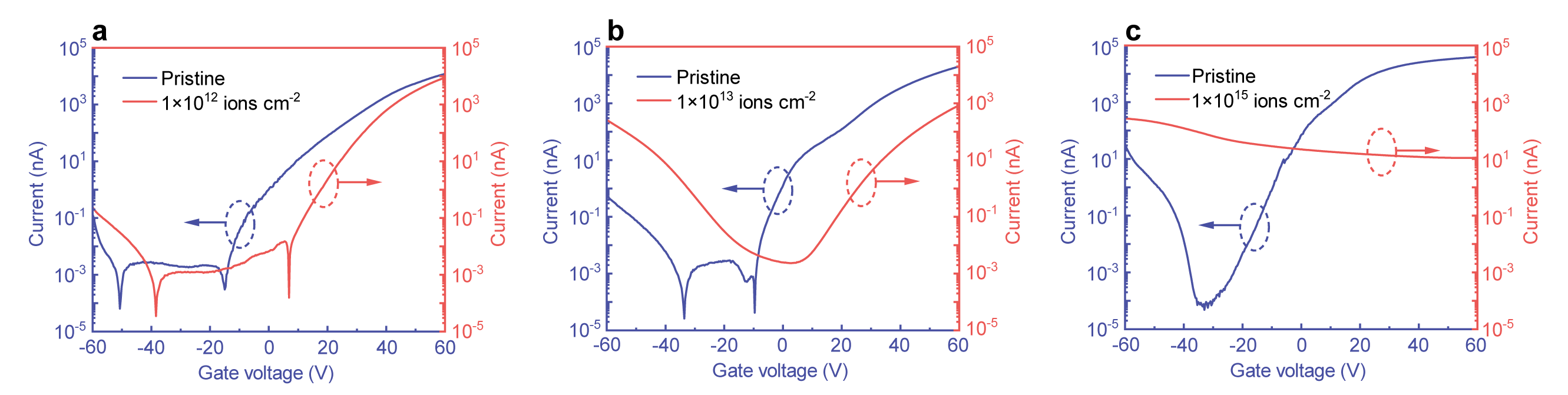


**Fig. S8** Transfer characteristic curves comparation between WS_2_ (blue curve) and N-WS_2_ (red curve) FETs with implantation doses of **(a)** 1×10^12^ ions cm^-2^, **(b)** 1×10^13^ ions cm^-2^, and **(c)** 1×10^15^ ions cm^-2^ at the bias of 1 V in logarithmic scale

**
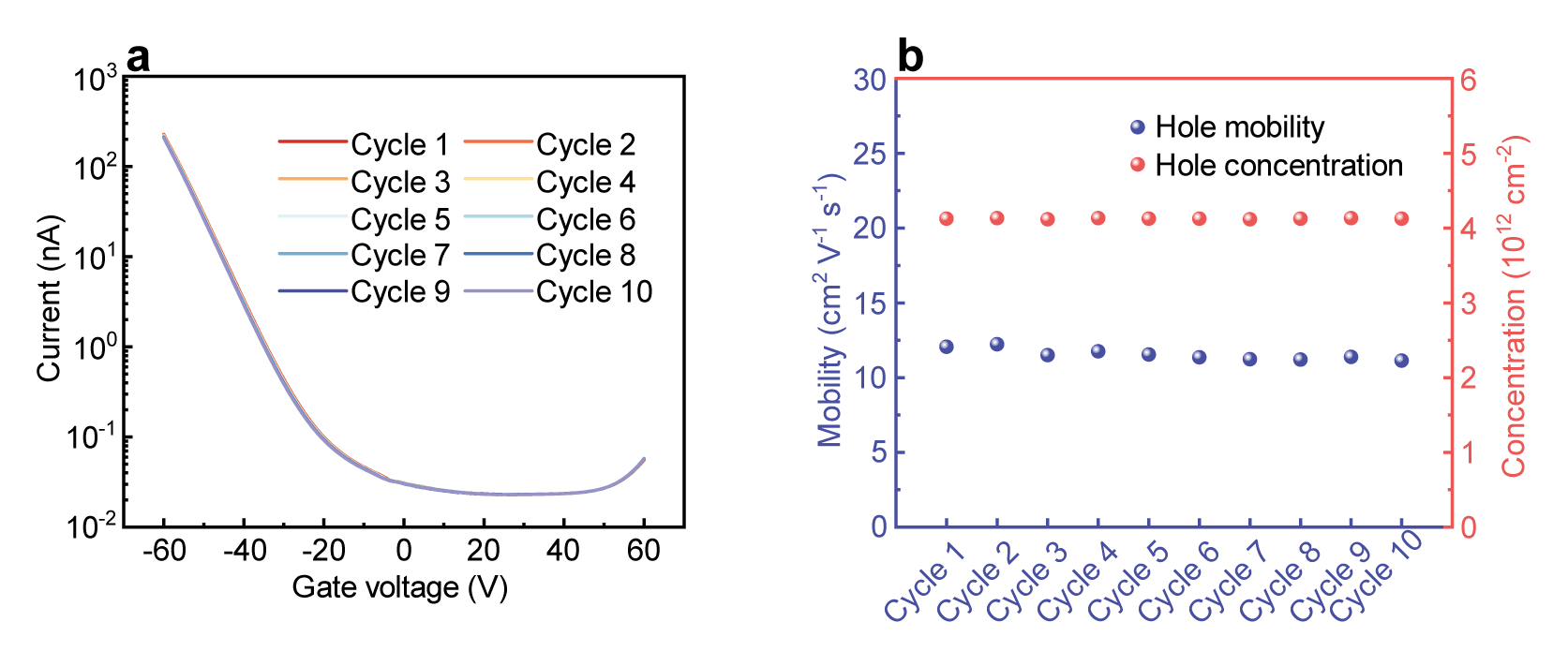
**

**Fig. S9** The device uniformity of N-WS_2_ FET on the same sample. **(a)** The transfer characteristic curves of N-WS_2_ on the same device for ten cycles. **(b)** The calculated hole mobility and carrier concentration of N-WS_2_ on the same device for ten cycles


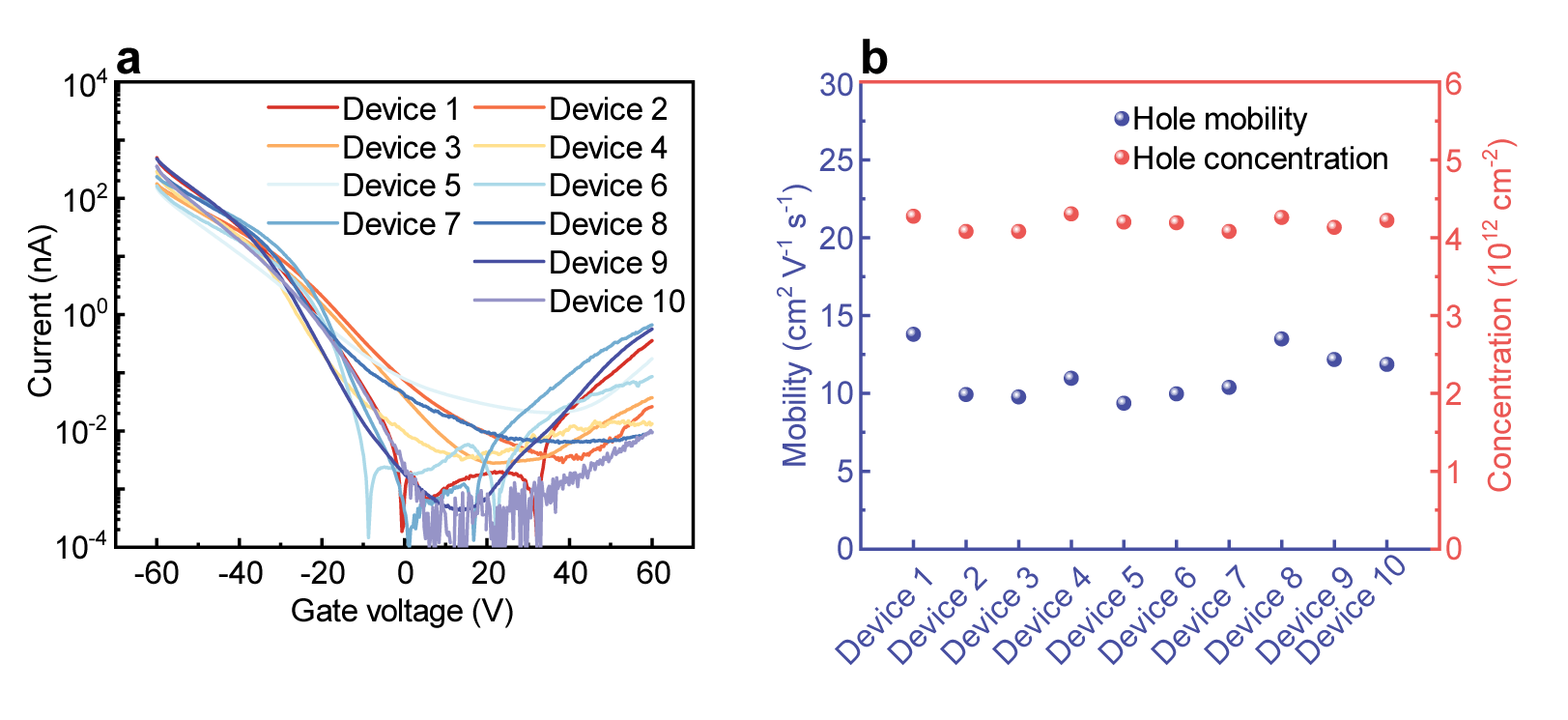


**Fig. S10** The device uniformity of N-WS_2_ FET for different devices. **(a)** The transfer characteristic curves of N-WS_2_ for different devices. **(b)** The calculated hole mobility and carrier concentration of N-WS_2_ for different devices


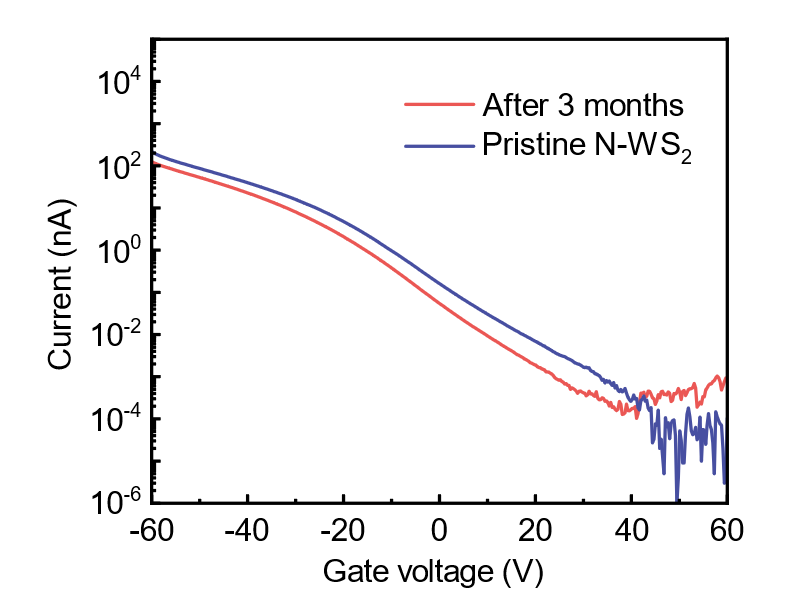


**Fig. S11** The difference of transfer characteristic curves of N-WS_2_ FET before and after three months


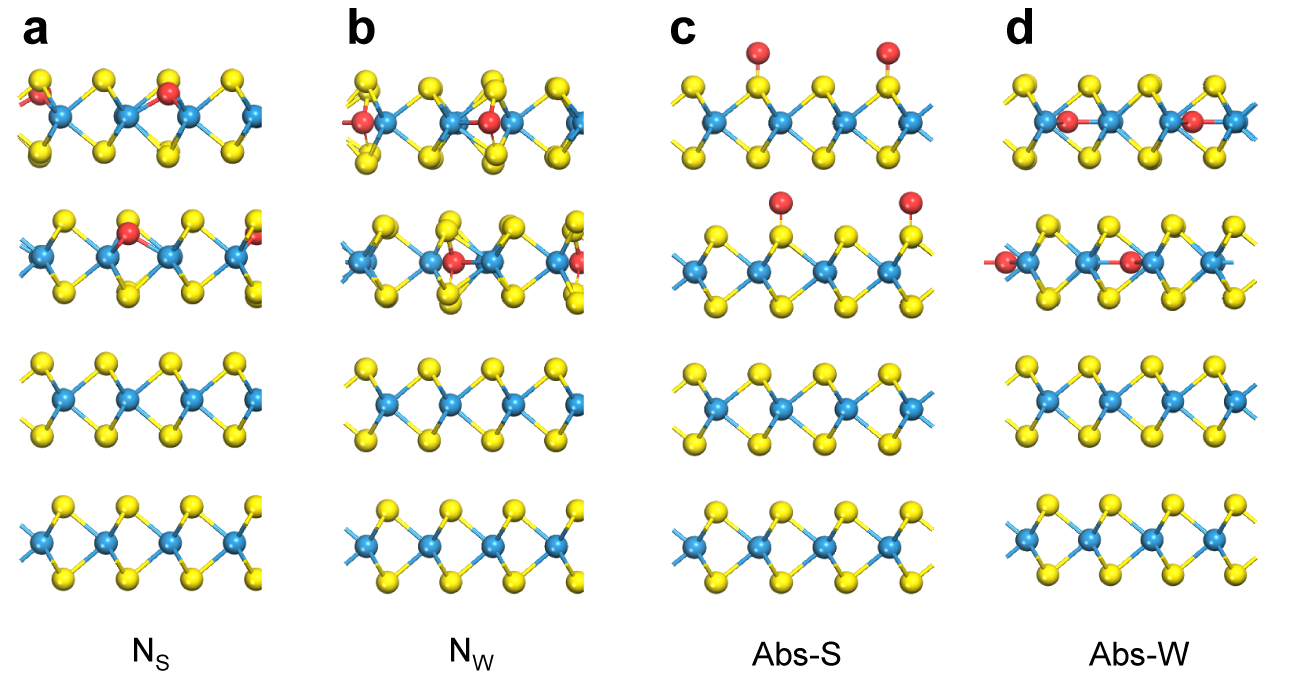


**Fig. S12** Atomic configurations of four possible N embedding sites in WS_2_. **(a)** Substitutional N site at S site (N_S_). **(b)** Substitutional N at W site (N_W_). **(c)** N adatom above S atom (Abs-S) (Interlayer adsorption). **(d)** N adatom above W atoms (Abs-W) (Interstitial adsorption). The yellow, blue and red balls represent S, W and N atoms, respectively


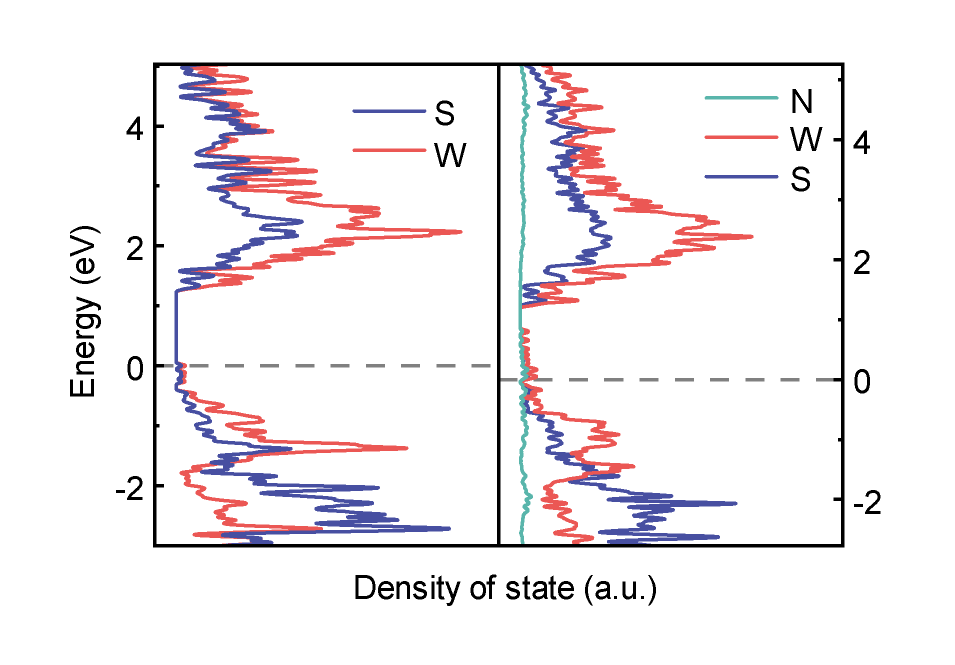


**Fig. S13** Element PDOS of pristine WS_2_ and N-WS_2_

**
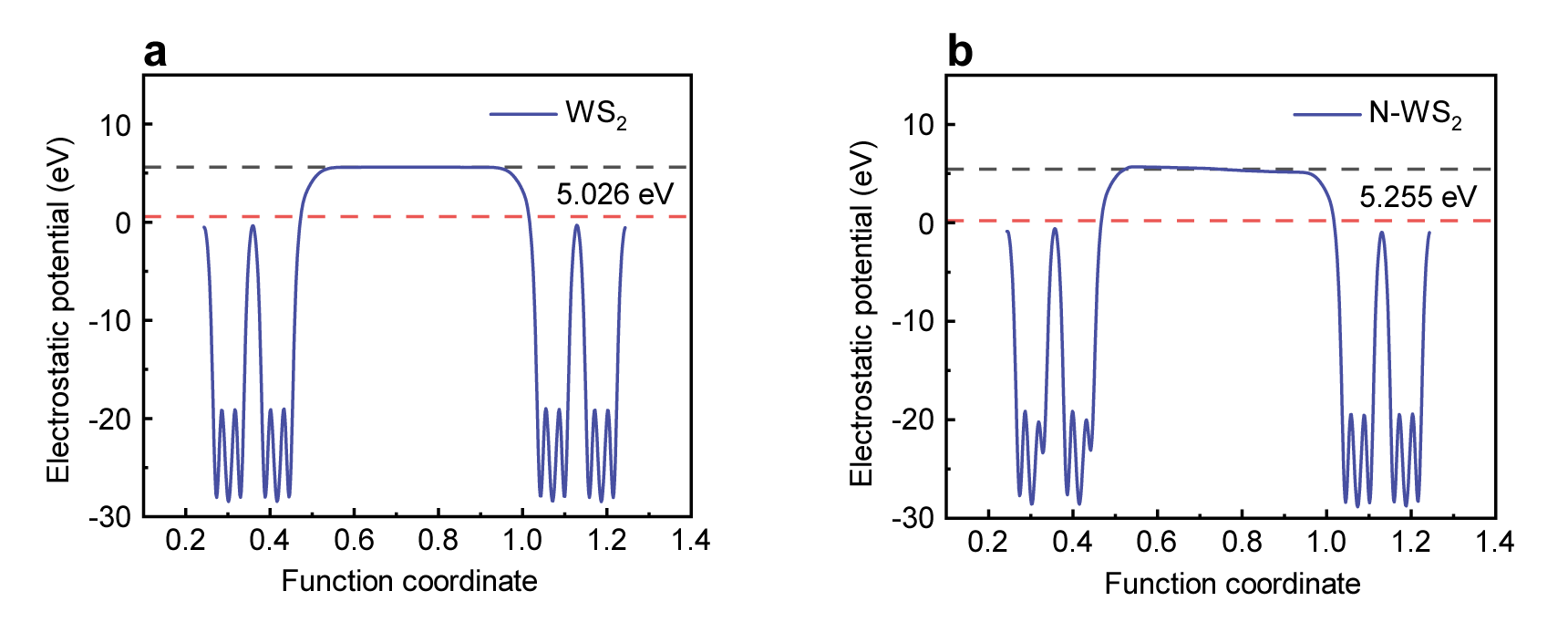
**

**Fig. S14** DFT calculated work functions of **(a)** pristine WS_2_ and **(b)** N-WS_2_


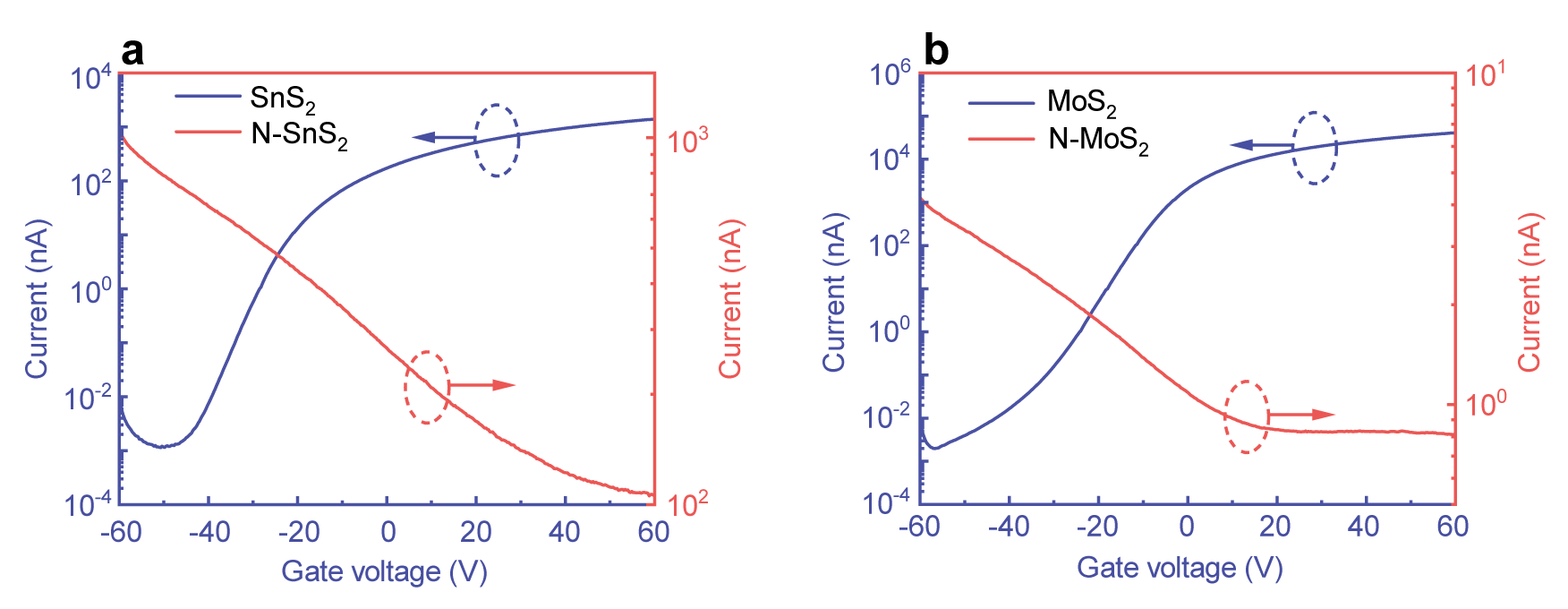


**Fig. S15** Transfer characteristic curves of **(a)** pristine SnS_2_ and N-SnS_2_ FET, **(b)** pristine MoS_2_ and N-MoS_2_ FET at the bias of 1 V in logarithmic scale


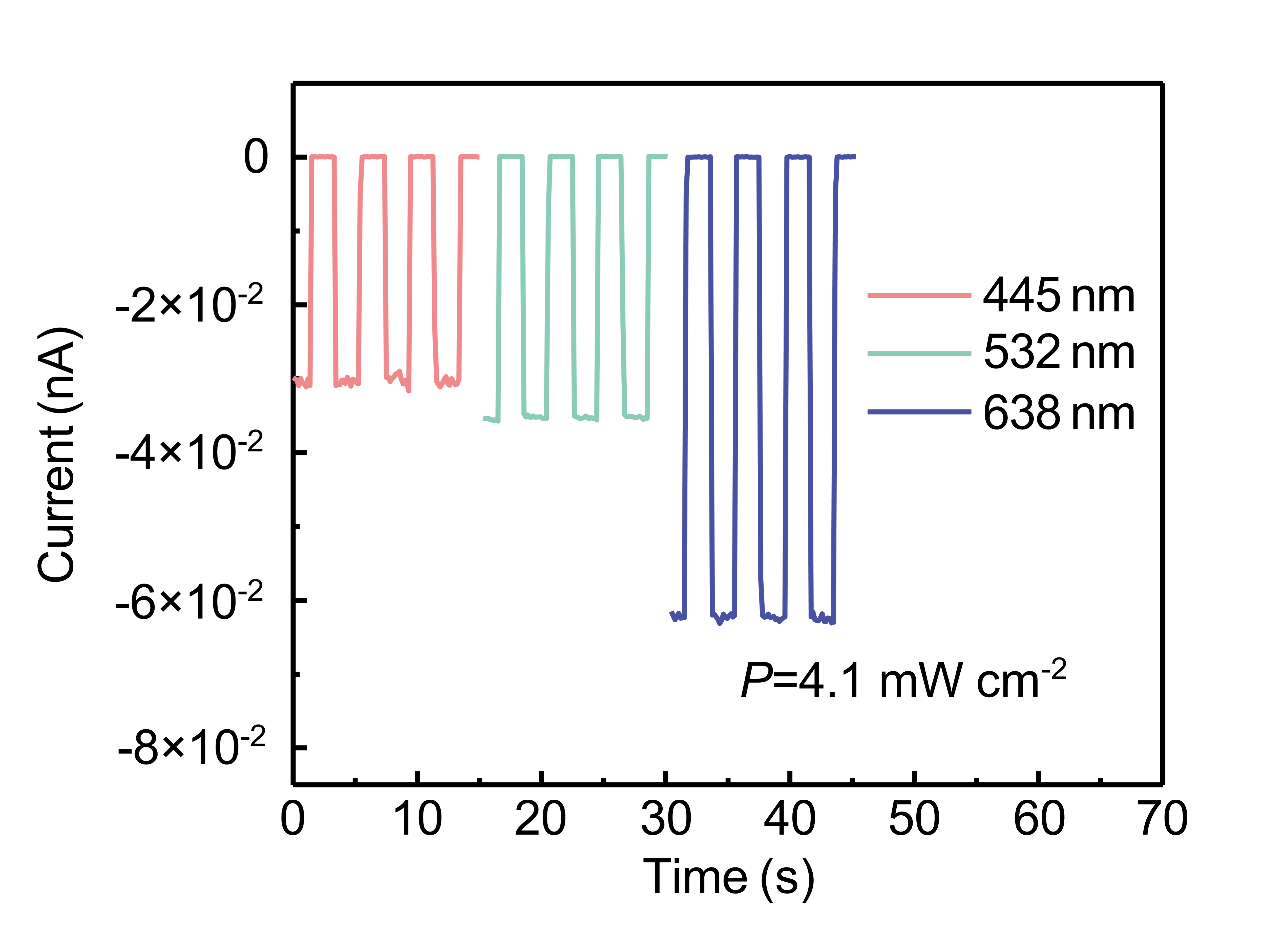


**Fig. S16** Transient photo-response of p-n junction under different wavelength illumination of 445 nm, 532 nm and 638 nm with the same power density of 4.1 mW cm^-2^


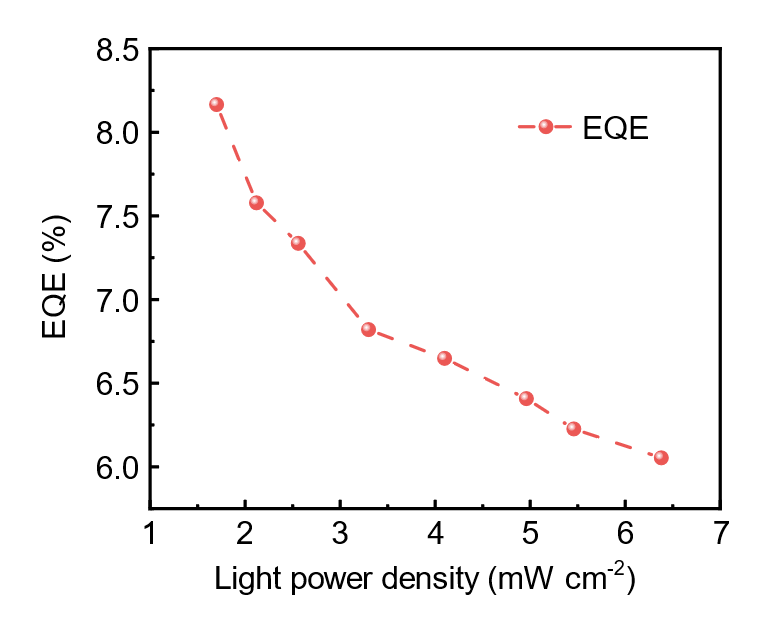


**Fig. S17** Light power dependence relation curves of *EQE*


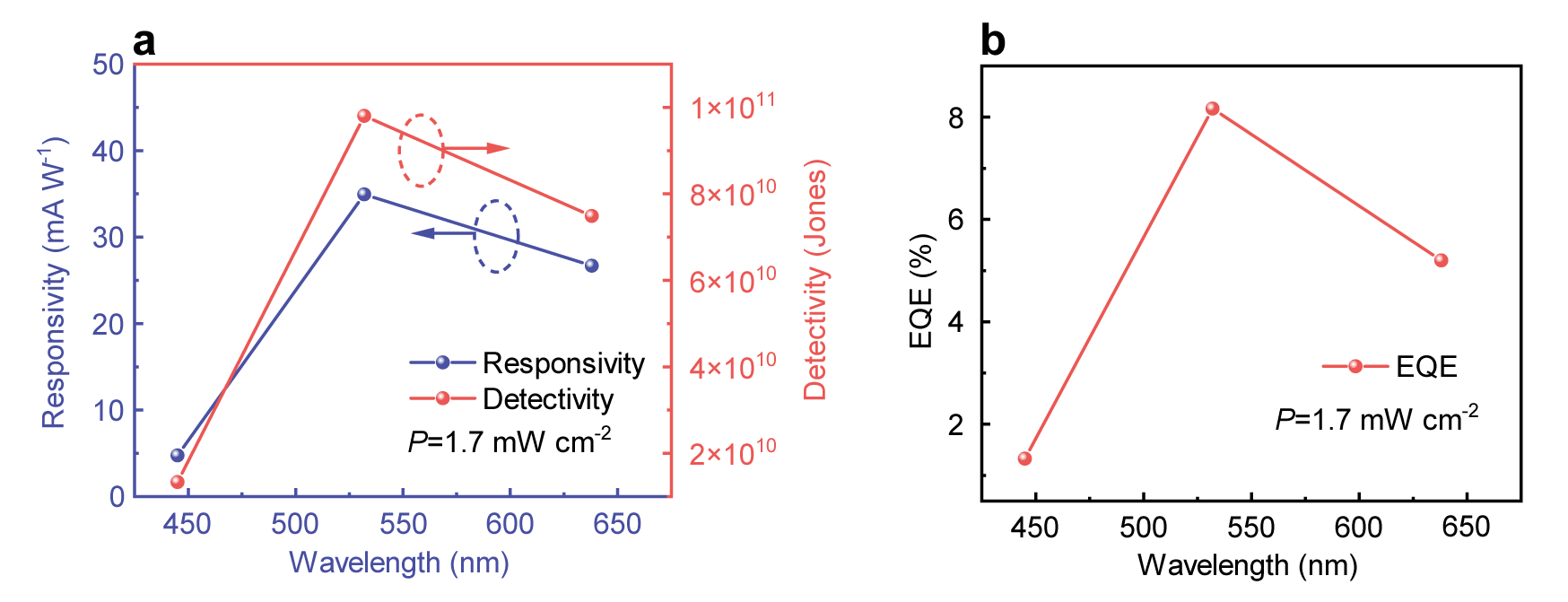


**Fig. S18** Wavelength dependence relation curves of **(a)** responsivity and detectivity and **(b)** *EQE*


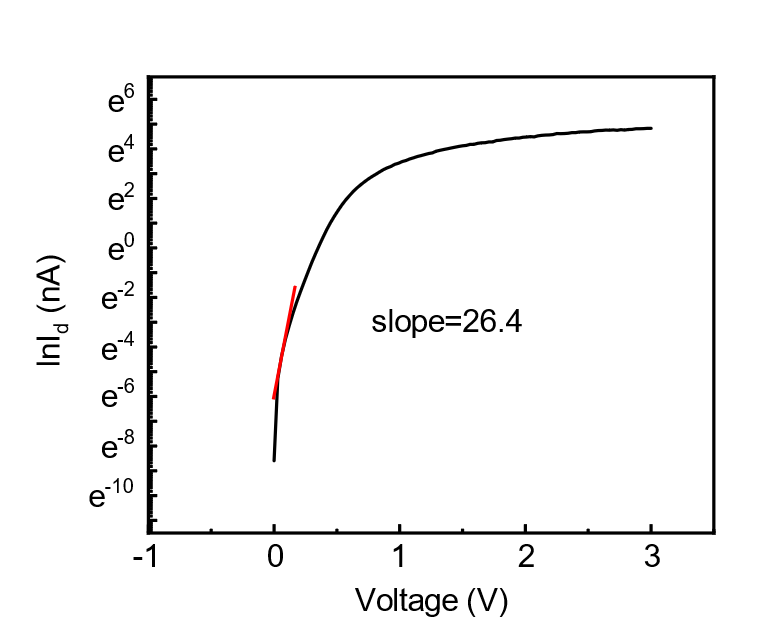


**Fig. S19** The relationship curve between *lnI_d_* and *V_d_*


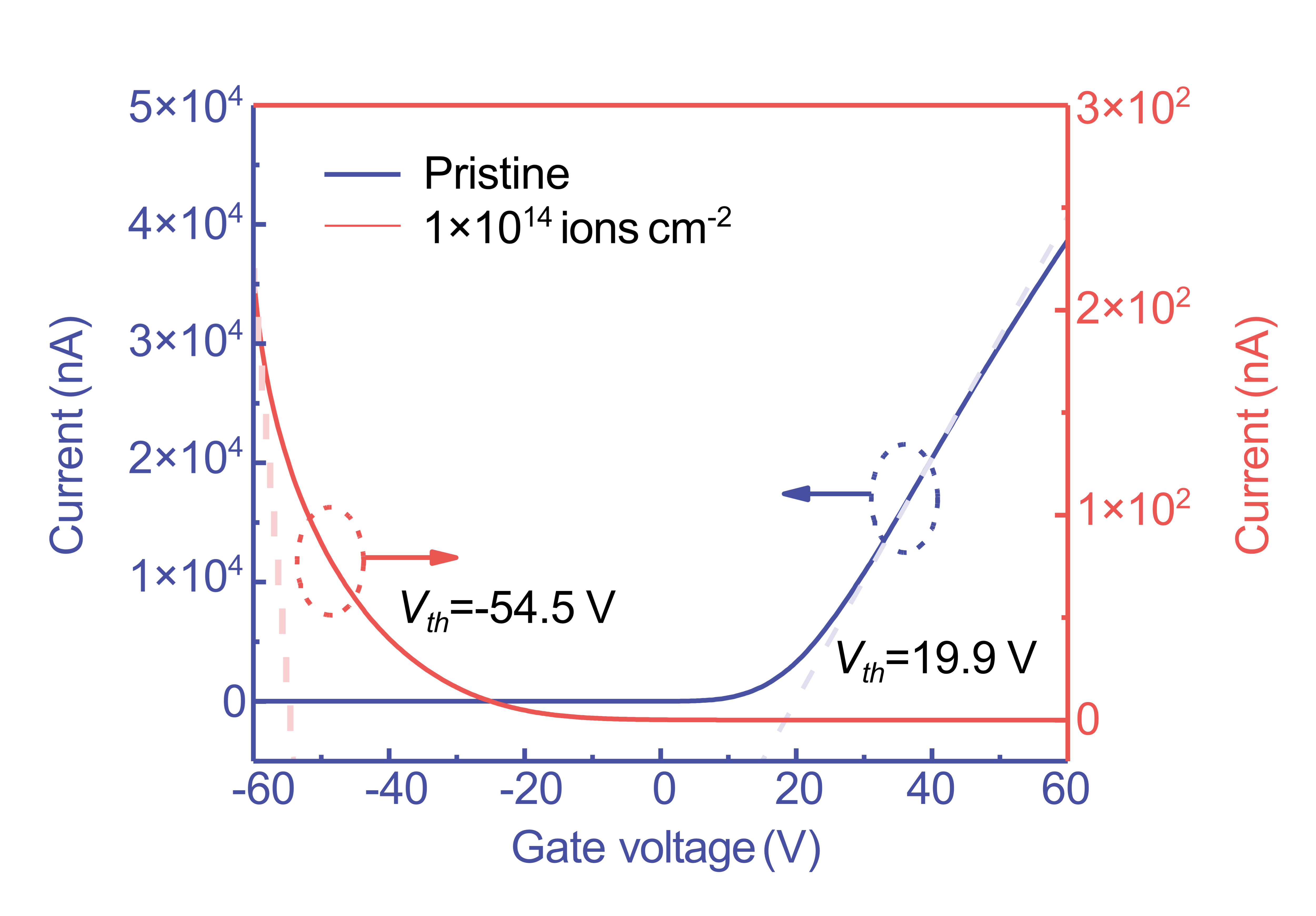


**Fig. S20** Transfer characteristic curves of WS_2_ and N-WS_2_ (with implantation dose of 1×10^14^ ions cm^-2^) FET at the bias of 1V

**Note S1. Calculation of the contact potential difference**

The work function of the samples can be expressed by the contact potential difference (*V_CPD_*) between the probe tip and the surface of the sample^1^. It can be calculated as:

$V_{CPD}=\frac{\varphi_{sample}-\varphi_{probe}}{-e}$ (1)

where *φ_sample_*, *φ_probe_* and e are the work functions of sample and probe tip and the elementary charge, respectively.

**Note S2. Calculation of the rectifier ideality factor**

In order to quantitatively evaluate the property of diode, the ideality factor (*n*) is extracted from the Shockley diode equation^2^, which provided the I-V characteristic of diode under forward or reverse bias. It can be calculated as:

$I_{d}=I_{s}[\exp\left( \frac{qV_{d}}{nkT} \right)-1]$ (2)

where *I_d_* and *I_s_* are respectively defined as forward and reverse bias saturation current, *q*, *k*, *T* and *V_d_* represent elementary charge, Boltzmann constant, absolute temperature and bias, respectively.

When *V_d_* is large than $\frac{3kT}{q}$, $\exp\left( \frac{qV_{d}}{nkT} \right)$ is much greater than 1, the Equation 2 can be simplified as:

$I_{d}=I_{s}\exp\left( \frac{qV_{d}}{nkT} \right)$ (3)

Logarithms are taken from both sides of the Equation 3, the following Equation is obtained:

${lnI}_{d}={lnI}_{s}+\frac{qV_{d}}{nkT}$ (4)

Equation 4 satisfies the linear equation, where $\frac{q}{nkT}$ is the slope, while the slope of *lnI_d_* about *V_d_* can be obtained by linear fitting. Consequently, the ideal factor *n* can be extracted from the slope, that is:

$\frac{dlnI_{d}}{dV_{d}}=\frac{q}{nkT}$ (5)

Firstly, the data of the output characteristic curve on p-n junction at gate voltage of 60 V is extracted. Then the *I_d_* is logarithmically processed, and the relationship curve between *lnI_d_* and *V_d_* is obtained. As shown in the Fig. S19, by calculating the slope, $\frac{dlnI_{d}}{dV_{d}}$ is obtained, which is about 26.4. According to the Equation 5, the value of *n* is about 1.46.

**Note S3. Calculation of threshold voltage and carrier concentration.**

The threshold voltage is obtained by finding the gate voltage axis intercept of the linear extrapolation of the transfer characteristic curve at its maximum slope point.

The carrier concentration is calculated by the formula^3^:

$n(p)=\frac{C_{g}|V_{g}-V_{th}|}{e}$ (6)

where *e* = 1.6 × 10^−19^ C, *C_g_* = 1.2 × 10^−8^ F cm^-2^ for 300 nm SiO_2_, *V_th_* and *V_g_* are expressed as threshold voltage and gate voltage, respectively.

As extracted from Fig. S20, the threshold voltage is about 19.9 V for WS_2_ and -54.5 V for N-WS_2_ at implantation dose of 1×10^14^ ions cm^-2^. After calculating, the electron and hole concentration at zero gate voltage are approximately 1.49×10^12^ cm^-2^ and 4.09×10^12^ cm^-2^, respectively.

**Note S4. Calculation of carrier mobility.**

The carrier field-effect mobility (*μ*) is calculated by the formula^1^:

$\mu=\frac{Lg_{m}}{WC_{g}V_{ds}}$ (7)

where $g_{m}=\frac{dI_{ds}}{dV_{g}}$ is the transconductance, *L*, *W* and *V_ds_* are the length, width of the channel and the source-drain voltage.

The *g_m_* of N-WS_2_ FET in Fig. 4e is 4.02×10^-8^ S (*V_g_*=-60 V). At the implantation dose of 1×10^14^ ions cm^-2^, the hole mobility at bias of 1 V is obtained about 12.16 cm^2^ V^-1^ s^-1^.

**References:**

1 Li, Z. *et al.* Universal p-Type Doping via Lewis Acid for 2D Transition-Metal Dichalcogenides. *ACS Nano* **16**, 4884-4891 (2022).

2 Wang, H. *et al.* Approaching the External Quantum Efficiency Limit in 2D Photovoltaic Devices. *Advanced Materials.* **34**, 2206122 (2022).

3 Tang, B. *et al.* Direct n- to p-Type Channel Conversion in Monolayer/Few-Layer WS_2_ Field-Effect Transistors by Atomic Nitrogen Treatment. *ACS Nano* **12**, 2506-2513 (2018).
